# Disseminated Toxoplasmosis in an Allogeneic Hematopoietic Stem Cell Transplant Recipient: A Case Report and Review of the Current Literature

**DOI:** 10.7759/cureus.29185

**Published:** 2022-09-15

**Authors:** Aliyah Baluch, Guy Handley, Steven Ludlow, Austin Morrison, Ana Velez, Marcia C Rojas, Yanina Pasikhova

**Affiliations:** 1 Infectious Diseases, Moffitt Cancer Center, Tampa, USA; 2 Infectious Diseases, University of South Florida Morsani College of Medicine, Tampa, USA; 3 Pharmacy, Moffitt Cancer Center, Tampa, USA; 4 Clinical Pharmacy, Moffitt Cancer Center, Tampa, USA; 5 Infectious Disease, Moffitt Cancer Center, Tampa, USA

**Keywords:** sulfadiazine, pyrimethamine, double umbilical cord blood transplant, toxoplasmosis gondii, bone marrow transplant, cns toxoplasmosis

## Abstract

Reactivation infections in hematopoietic stem cell transplants are mitigated by prophylactic regimens. Despite high rates of exposure, morbidity and mortality secondary to toxoplasmosis are limited to subsets of patients such as immunocompromised persons. We describe the first case of disseminated toxoplasmosis in a double umbilical cord blood transplant recipient.

## Introduction

Reactivation of infections in hematopoietic stem cell transplants (HSCTs) is a risk mitigated by prophylactic regimens. Toxoplasmosis is a parasitic infection caused by *Toxoplasma gondii *that can lead to ring-enhancing brain lesions, pneumonia, or other atypical presentations in the immunosuppressed. *T. gondii* is a ubiquitous obligate intracellular parasite that commonly causes either clinical or latent infection in almost all mammals, which then is transmitted to humans either via undercooked contaminated meat or the feces of felines [1,2]. It is estimated that 11% of the United States population aged six years and older have been infected with toxoplasmosis, and, internationally, the rates may be as high as 95% [1]. Despite high rates of exposure, significant morbidity and mortality secondary to toxoplasmosis are typically limited to certain subsets of patients such as fetuses, infants, and the immunocompromised [1-3]. Although the presentation may vary across at-risk groups, in HSCT recipients, it tends to be disseminated with a focus on the central nervous system (CNS), lungs, and heart and as a result of reactivation of previously seropositive individuals [3].

At our facility, screening for toxoplasmosis serology is the standard of care in all HSCT recipients during pre-transplant evaluation. In patients with positive serology, post-allogeneic HSCT (alloHSCT) transplant prophylaxis includes sulfamethoxazole/trimethoprim (SXT) which has activity against *T. gondii* that traditionally also co-suppresses *Pneumocystis jirovecii* [4].

Unlike in the human immunodeficiency virus (HIV) population where explicit guidelines are available for prophylaxis against toxoplasmosis, in the HSCT field, there are ongoing discussions regarding the optimal agent, duration, or subset of patients for suppression. Patients who receive high-intensity chemotherapy and immunosuppression are at high risk for reactivation of infections such as toxoplasmosis in the absence of appropriate prophylaxis. Patients undergoing double umbilical cord blood transplants (DUCBT) are at high risk. We describe what is thought to be the first case of disseminated toxoplasmosis in an adult recipient of a DUCBT who fully recovered after treatment with triple-drug therapy.

## Case presentation

A 37-year-old Hispanic male presented to a tertiary cancer center complaining of fevers up to 39.5°C, light-headedness, nausea, fatigue, and generalized malaise for the last 24 hours. Due to his primary disease of acute myelogenous leukemia (AML), the patient was day 60 post-DUCBT with the conditioning regimen of fludarabine (200 mg/m^2^), cyclophosphamide (50 mg/kg) and total body irradiation (TBI) (200 cgy).

Pertinent infectious history prior to transplant included an episode of hepatosplenic candidiasis with *Candida tropicalis* resulting in nodular pneumonia and hepatic and splenic lesions, which was treated with voriconazole and micafungin. Pertinent positive pre-transplant serologies included herpes simplex virus (HSV), varicella-zoster virus (VZV), and toxoplasmosis immunoglobulin G (IgG). In addition, the patient was noted to have residual pulmonary and hepatosplenic lesions and was continued on voriconazole and micafungin combination at the time of transplantation. Additional antimicrobial prophylaxis included doxycycline, ciprofloxacin, and valacyclovir per institutional protocol. The patient’s post-transplant course was complicated by human herpes virus 6 (HHV-6) viremia, which was treated with foscarnet, and acute graft-versus-host disease (aGVHD) of the skin and gastrointestinal (GI) tract, which was treated with oral beclomethasone, oral budesonide, and topical triamcinolone.

The patient was discharged with close follow-up. On transplant day 62, the patient presented with a headache with frontal region pressure, nausea, and rash (chest, trunk, and bilateral upper extremities) developing over two days. He reported fatigue and a fever of 39.5°C, which failed to respond to acetaminophen. Medications at the time of presentation included sirolimus 2 mg daily three times per week, 1 mg daily on the remaining days, and tacrolimus 1 mg twice per day for GVHD prophylaxis; voriconazole for antifungal prophylaxis; valacyclovir for HSV and VZV prophylaxis; and nebulized pentamidine 300 mg every 28 days for *P. jirovecii* prophylaxis. *P. jirovecii* prophylaxis, which started on day 28 as per protocol, was changed from pre-planned SXT to pentamidine one month prior due to poor count recovery and concern that the drug may be contributing to pancytopenia.

Infectious Diseases was consulted. The patient was febrile at 38.4°C with a heart rate of 125 beats per minute. On examination, a remarkable finding was a maculopapular rash over the anterior torso. His weight was 77 kg, and his height was 173 cm. His social history was notable for being born in Brazil, living in New Orleans, then mostly in Miami, working for Homeland Security and the Navy, and traveling to various places including Haiti. There was no history of tuberculosis, malaria, or dengue fever. He smoked a pack of cigarettes per month for two years, which he quit 12 years prior, and he denied drug or alcohol use.

Laboratory findings were significant for pancytopenia, white blood cell count of 1.61 k/µL (4-10.9 k/µl) with an absolute neutrophil count of 1.08 k/µL (1.8-7.8 k/µL), hemoglobin and hematocrit were 10 g/dL (11.4-15 g/dL) and 29.5% (34.3-45.4%), respectively, and the platelet count was 17 k/µL (143-382 k/µL). Blood chemistries were all within normal limits. Blood and urine cultures, respiratory viral panel, and computed tomography (CT) of the thorax, abdomen, and sinuses were obtained. Empiric antimicrobial therapy with cefepime 2 g intravenously every 12 hours and vancomycin 1,250 mg intravenously every eight hours was initiated. The patient was admitted for further evaluation.

CT of the thorax and abdomen showed improvement in the patient’s pulmonary and liver nodules compared to the prior examination. CT of the sinus was pertinent for mild sinusitis. Blood and urine cultures showed no growth, and the respiratory viral polymerase chain reaction (PCR) panel was negative. Despite 72 hours of empiric broad-spectrum antimicrobials and negative workup, the patient continued to worsen. His temperature increased to 39.4°C and he complained of worsening headaches.

**Figure 1:**
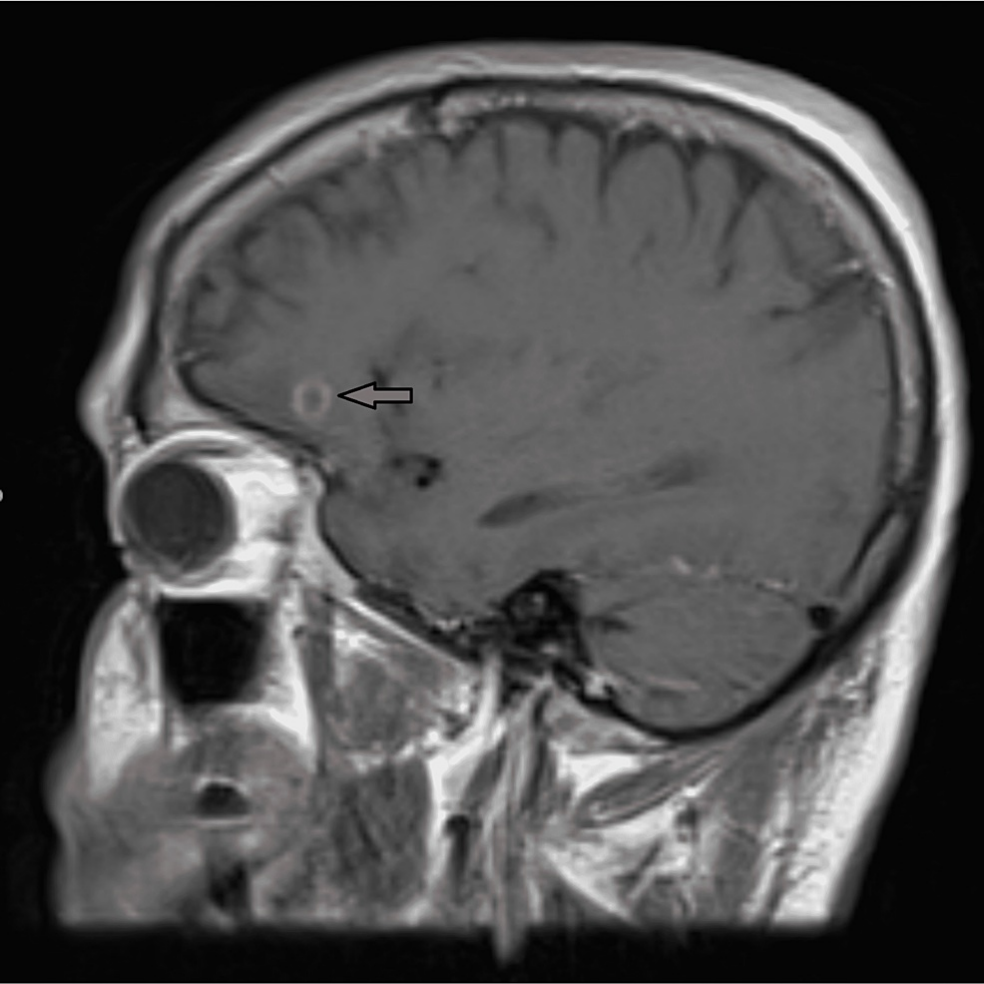
Sagittal T1-weighted magnetic resonance imaging of the initial 0.7 cm ring-enhancing lesion in the left inferior frontal cortex.

Plasma viral studies, including HHV-6, Epstein-Barr virus (EBV), and cytomegalovirus (CMV) by PCR were negative or detectable but not quantifiable. Magnetic resonance imaging (MRI) of the brain revealed a 0.7 cm ring-enhancing lesion in the left inferior frontal cortex with a small amount of vasogenic edema (Figure 1). A presumptive diagnosis of cerebral toxoplasmosis was made leading to the addition of therapy with pyrimethamine 75 mg daily, sulfadiazine 1,500 mg every six hours, and leucovorin 20 mg daily on hospital day four. Blood was drawn and a lumbar puncture was performed to run a *T. gondii* PCR. The cerebrospinal fluid (CSF) and plasma PCRs were both positive for the protozoan parasite. Despite standard first-line therapy with pyrimethamine and sulfadiazine, the patient became hemodynamically unstable with a blood pressure of 95/50 mmHg and tachycardia combined with respiratory distress and hypoxia. On hospital day eight, the patient required intubation and mechanical ventilation.

A bronchoscopy and bronchoalveolar lavage (BAL) was performed. The BAL was sent for standard and immunosuppressed panels, including bacterial, fungal, acid-fast bacillus (AFB) stains, and cultures for *P. jirovecii*, CMV, HSV, and *T. gondii*. The pertinent positive finding from the BAL was a positive PCR for *T. gondii*. Combining the patient’s clinical signs and symptoms with the positive PCR results from CSF, blood, and BAL, he was diagnosed with disseminated toxoplasmosis. Despite 18 days of therapy with pyrimethamine and sulfadiazine combined with a reduction in immunosuppression, the patient remained intubated, febrile, and failed to demonstrate clinical improvement. A clinical decision was made between the Infectious Diseases, Pharmacy, and Bone Marrow Transplant teams to add clindamycin 600 mg intravenously every eight hours to his current toxoplasmosis treatment.

**Figure 2:**
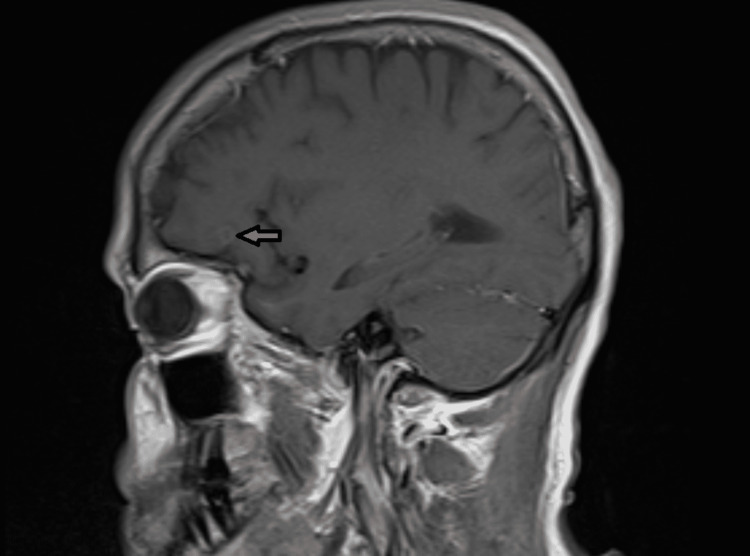
Sagittal T1-weighted magnetic resonance imaging demonstrating interval improvement in the size and intensity of the ring-enhancing lesion in the left inferior frontal cortex.

After seven days of triple-drug therapy, the patient began to tolerate weaning trials with improving respiratory status. On hospital day 30, a repeat MRI of the brain showed an interval slight decrease in the size of the ring-enhancing lesion in the left frontal lobe and a concomitant decrease in surrounding vasogenic edema (Figure 2). Ten days after clindamycin was added to the antimicrobial regimen, the patient was extubated. He completed a total of 21 days of triple-drug therapy for disseminated toxoplasmosis and was ultimately discharged home on hospital day 40.

The patient was discharged with the goal of completing six weeks of induction therapy with pyrimethamine 75 mg daily, sulfadiazine 1,500 mg every six hours, and leucovorin 20 mg daily, followed by maintenance therapy of pyrimethamine 25 mg daily, sulfadiazine 1,000 mg every 12 hours, and leucovorin 20 mg daily, on which the patient was to remain until off immunosuppression and a CD4 count over 200 for at least six months. He was also advised to undergo a repeat MRI of the brain to further guide the duration of therapy. The patient continued secondary prophylaxis for almost two years, which was discontinued once there was complete resolution of the brain lesion on imaging, discontinuation of all immunosuppression, and CD4 count over 200 for six months. Despite the severity of his disease and the complicated clinical course, the patient does not have any permanent neurological sequelae and is currently in remission.

## Discussion

Toxoplasmosis reactivation or new-onset infection is a relatively rare infectious complication of HSCT recipients occurring in 0.3-8% of patients, typically 30 days post-HSCT in 90% of the cases [3-9]. Mele et al. compiled 110 published cases of toxoplasmosis infections in HSCT recipients which revealed that 63% of patients were male, 77% of recipients had some degree of GVHD, and 88% of recipients had a positive serology prior to HSCT. It was also found that 48% of cases were isolated cerebral toxoplasmosis whereas 76% of cases of disseminated infection also included the CNS [3]. The median days post-HSCT at the onset of toxoplasmosis was 62 (range: 1-689). The patients diagnosed with disseminated toxoplasmosis had 10.8 times the risk of having the diagnosis made post-mortem than those diagnosed with cerebral toxoplasmosis [3]. One of the main risk factors predisposing double cord recipients to earlier toxoplasmosis reactivation compared to other HSCT recipients is related to the higher degree and duration of immunosuppression related to the transplantation [4,10].

*T. gondii* has two distinct life cycles: an infective cycle and a diagnostic cycle. Domestic cats are the definitive host for *T. gondii* and shed unsporulated oocysts in their feces. After the oocysts have sporulated in the environment, they are taken up by intermediate hosts such as rodents and birds. Other cats become infected by ingesting the intermediate hosts and their tissue cysts. The second life cycle, the asexual phase, occurs when non-definitive hosts, such as humans, are infected. This can be by accidental ingestion of oocysts [1] or by acute transmission via organ transplantation or blood transplantation such as an HSCT [3]. Lastly, toxoplasmosis may be transferred from the mother to the fetus [1].

Bautista et al. calculated the rate of toxoplasmosis to be as high as 6% in cord blood transplant recipients and 0.2% in all other types of allogeneic HSCT recipients [11]. In the literature review of the nine cases with cord blood transplants, five (56%) of the recipients presented disseminated disease and all five died due to toxoplasmosis, where three of the five patients were diagnosed on autopsy. Only one patient received pyrimethamine and sulfonamides as a treatment for more than four days [11].

Diagnosing toxoplasmosis infection in HSCT recipients continues to be difficult as the symptoms tend to be vague. These symptoms include fever, pneumonitis, myocarditis, meningitis, headache, brain abscess, lymphadenopathy, hepatosplenomegaly, chorioretinitis, hepatitis, pancytopenia, or disseminated disease [8]. The use of toxoplasmosis PCR of blood or CSF may aid in timely diagnosis but there is no data supporting a correlation with decreased mortality. The most important factors influencing the outcomes are the site of infection, the presence of symptoms at onset, and the conditioning regimen. Because these symptoms are nonspecific, early diagnosis remains a challenge ultimately leading to disseminated cases with unacceptable mortality. Disseminated toxoplasmosis with multiple organ involvement is frequently reported post-mortem on autopsy [3].

The best-evaluated first-line therapy against toxoplasmosis is the combination of pyrimethamine (a dihydrofolate reductase inhibitor) and sulfonamides. This combination is well absorbed in the small bowel and crosses the blood-brain barrier. The combination of pyrimethamine and clindamycin was proven to be almost as effective and is widely used as second-line therapy. However, the combination of clindamycin, pyrimethamine, and leucovorin exposes the patient to myelosuppression [11].

This clinical case was unique as a double cord blood transplant recipient developed disseminated toxoplasmosis and was diagnosed by PCR of blood, CSF, and bronchoalveolar lavage. The brain lesions were also indicative of cerebral toxoplasmosis. Once the MRI reported brain lesions, the patient started first-line therapy for toxoplasmosis as the guidelines recommend. Unfortunately, the patient continued to deteriorate while on mechanical ventilation. Once clindamycin was added to pyrimethamine and sulfadiazine, the patient showed clinical improvement without long-term neurologic sequelae.

## Conclusions

Disseminated toxoplasmosis in cord blood transplant recipients continues to be one of the most fatal and severe opportunistic infections. It is important to note that the infection can be prevented by serology screening prior to transplant, through proper prophylaxis, and by being aware of toxoplasmosis as a possible diagnosis in cord blood transplant recipients. Regarding treatment, it is important to highlight the outcome of our patient. The complete lack of neurological symptoms after triple-drug therapy is quite astounding. These findings illustrate a need for continued research in this area.
